# The influence of temperature on river fly phenology based on historic records

**DOI:** 10.1007/s00484-026-03321-2

**Published:** 2026-09-17

**Authors:** Tim H. Sparks, Annette Menzel

**Affiliations:** 1 Institute of Advanced Study and TUM School of Life Sciences (Ecoclimatology), Technical University of Munich, Munich, Germany; 2 Poznań University of Life Sciences, Poznań, Poland; 3 Museum of Zoology, University of Cambridge, Cambridge, UK

**Keywords:** Caddis fly, Chalk streams, Emergence, Fly fishing, Mayfly

## Abstract

First appearance dates of three river flies (Mayfly, two caddis flies: Grannom and Welshman’s Button) for 1834–1931 were abstracted from the records of a chalk river fly fishing club in southern UK. First appearance dates of the terrestrial invertebrates Mole Cricket and Glow-worm were also abstracted. Appearance dates were related to monthly mean air temperatures by multiple regression. For all but Mole Cricket, modest but significant advances in phenology were detected in years with warmer temperatures; typically of the order of 3–6 days/°C. However, limited meteorological data were available for this historic period and we suggest that improved models require additional and more fine scale local weather and environmental data.

## Introduction

The inherent need for healthy rivers is recognised by the EU Water Framework Directive and pollution-sensitive invertebrates are excellent bioindicators of river health (Hawkes 1998). However, the phenology of such species is understudied and largely represented by data sets of limited duration (Woods et al. 2022). Short-term (e.g. Finn et al. 2022) or experimental studies (e.g. McCauley et al. 2015) dominate published research on the emergence timing of freshwater (macro)invertebrates with a terrestrial component to their lifecycle. There are a small number of exceptions (e.g. Elliott 1996; Hassall et al. 2007), but Woods et al. (2022) acknowledge the main problem of a scarcity of data. In contrast to terrestrial species, there is consequently also a lack of knowledge on the phenological responses of (mostly) ectotherm freshwater organisms to environmental stressors (Thackeray et al. 2010), such as changes in temperature, hydrology and riparian shading.

The River Test in Hampshire is one of the UK’s foremost trout fishing rivers. The Houghton Fishing Club was founded here in 1822, and it is the world’s oldest and possibly most exclusive fly fishing club. Two publications (Maxwell 1908; Page 1932) chronicle the first century of its existence. These contain the first appearance dates of three aquatic invertebrates (one mayfly and two caddis flies) as well as of two (now rare) terrestrial invertebrates (Mole Cricket and Glow-worm). It is the uniqueness of these records that merit an investigation of the influence of temperature on first appearance dates of some freshwater macroinvertebrates previously underrepresented in phenological research.

The Test was described contemporarily, although perhaps in flowery language, by Dewar (1899:89–106) “The Test is a pure chalk stream…abounding with splendid trout, and in some places with grayling of great size… above and below [Stockbridge] the water is fished by the Houghton Club”. Dewar (1899) further stated that the river wasn’t affected by water extraction or badly by pollution. As a chalk stream, water temperatures of the Test should be largely driven by groundwater in addition to the influences of substrate type and shading. In spring, monthly water temperatures in UK chalk streams are largely a linear function of mean air temperature, with air temperatures lower than water temperatures when below 15 °C (Environment Agency 2022).

Fly fishing for trout is a very popular, although expensive, hobby. Consequently, a keen interest is taken of when specific artificial flies/patterns, mimicking real river flies, are suitable. Many of these river flies have longer than an annual life cycle, with a disproportionately short time as aerial flying adults, the remainder below water as earlier developmental stages. Despite the intense interest in emergence dates, there are relatively few publications based on long-term annual records of these insects (e.g. Elliott 1996). We here examine whether temperature drives first appearance/emergence dates of five invertebrates.

## Materials and methods

### Phenology data

Dates of first appearance of Grannom, Mayfly, “Caperer”, Mole Cricket and Glow-worm from the Houghton Fishing Club records 1825–1931 (Maxwell 1908; Page 1932) were abstracted, with the first invertebrate record in 1834. Dates were converted to day of the year (DOY, 1 = Jan 1st etc., taking into account Leap Years). Data were not recorded for all events every year.

The Grannom is most likely the caddis fly *Brachycentrus subnubilis*. Among the many species of Mayfly (Ephemeridae), the event recorded should be *Ephemera danica* (see also Reisinger et al. 2010). In recent literature, Caperer often refers to *Halesus* spp., e.g. *H. digitatus* or *H. radiatus* (Reisinger et al. 2010). However, Hutchinson (1925) clearly described how the Houghton Fishing Club’s Caperer is the (Black) Caperer or Welshman’s Button *Sericostoma personatum*. In addition, its reported emergence dates in May strongly support this identification, since *Halesus* spp. are August species. Mole Cricket is *Gryllotalpa gryllotalpa* and Glow-worm is possibly *Lampyris noctiluca*, one of two UK glow-worm species. For convenience only, all events are hereafter referred to as species.

### Life cycles

The three river flies spend a year or longer of their lifecycle under water, with an aerial imago component lasting from a few hours to a few days (or weeks) depending on species (Reisinger et al. 2010). The two caddis flies develop through egg, larval and pupal stages underwater before emerging as adults. Mayfly passes through egg and nymph stages underwater before emerging as a subimago, developing quickly into imago after a further moult.

The Mole Cricket spends most of its life underground and overwinters as larvae or young adults. After spring emergence, the mostly two-year life cycle includes nocturnal mating and egg laying underground. In their preferred grassland environments, soil temperature in their tunnel system of up to 1 m depth should not drop below 0 °C. Maturation of adult stages requires warming soils of 10–12 °C (Gazi 2018).

Glow-worms overwinter 2–3 times as larvae, followed by a brief pupation and then emerge as adults which are nocturnal from June to August.

### Temperature data

The nearest location that had monthly air temperature over the entire period was the Radcliffe Observatory in Oxford, 75 km north of Stockbridge. Monthly temperature data were downloaded from https://www.geog.ox.ac.uk/research/climate/rms/monthly-annual.html.

### Analysis

Mean dates of the five species were based on different sets of years, so a general linear model of first appearance date incorporating year and species, both as categorical variables, was run to generate adjusted least-square means of each species. To estimate temperature responses, first appearances (as DOY) of each species were separately regressed onto the three separate monthly temperatures in which the mean date for that species occurred and the two preceding months. Temporal synchrony between species in first appearance dates was assessed using Pearson correlations. Analysis was undertaken in Minitab22.

## Results

**Fig. 1:**
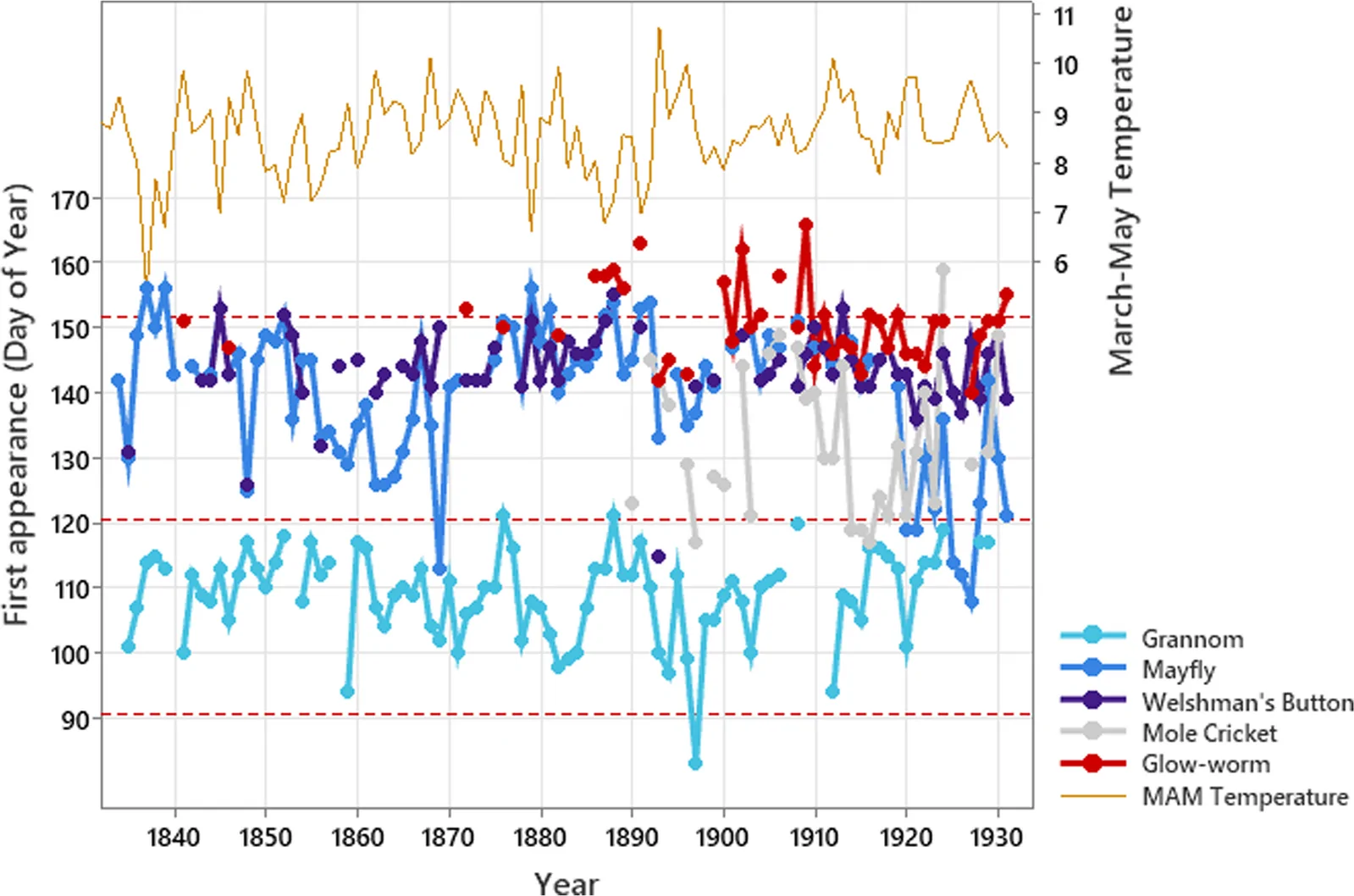
First appearance dates (day of the year) of the five invertebrates taken from the Houghton Fishing Club records in Hampshire, UK. Contiguous data are connected by lines. Horizontal dashed lines separate the months of March, April, May and June. Mean March-May (MAM) air temperatures from Oxford are shown for information at the top of the graph

A basic summary of the five species is given in Table 1 with a graphical representation in Fig. 1.

Between 33 and 89 years of data were available for each species. Arithmetic and adjusted means differed by no more than one day. The range of emergence dates for each species varied from 26 days for Glow-worm to 48 days for Mayfly. Grannom emerged about a month earlier than other species.

**Table 1:** For each species of the Houghton Fishing Club chronicles (1834–1931), the number of years of data, mean date, standard deviation, least squares adjusted mean date and extreme dates are summarised

	*n*	Mean date ± SD	Adjusted mean date	Earliest date	Latest date
Grannom	86	April 19 ± 6.8	April 18	Mar 24	April 30
Mayfly	89	May 20 ± 10.8	May 20	April 18	June 5
Welshman’s Button	66	May 23 ± 6.3	May 24	April 25	June 3
Mole Cricket	33	May 13 ± 12.0	May 14	April 27	June 7
Glow-worm	41	May 30 ± 5.9	May 30	May 20	June 15

The results of the regression models are shown in Table 2. All terms were included and the significance of individual terms is shown. All models, except for Mole Cricket, were significant. Summing the coefficients for each species suggests an advancing response of 3.6 days/°C for Grannom, 6.2 days/°C for Mayfly and 4.0 days/°C for Welshman’s Button. For Glow-worm the equivalent value is 4.8 days/°C and for Mole Cricket 3.4 days/°C, although the latter was not significant.

**Table 2:** Regression coefficients (days/°C) in the regression models for each species based on monthly mean temperatures in the month in which the mean date of appearance occurred and the preceding two months. Phenology data were taken from the Houghton Fishing Club records in Hampshire, UK and air temperatures from Oxford, UK

	Feb	Mar	Apr	May	*R* ^2^	*p*
Grannom	-0.6 *p* = 0.051	-1.7 *p* < 0.001	-1.3 *p* = 0.011		33.0	< 0.001
Mayfly		-1.8 *p* = 0.015	-2.8 *p* = 0.001	-1.6 *p* = 0.073	25.9	< 0.001
Welshman’s Button		-1.3 *p* = 0.010	-1.4 *p* = 0.015	-1.3 *p* = 0.026	27.3	< 0.001
Mole Cricket		-1.7 *p* = 0.283	-0.7 *p* = 0.749	-1.0 *p* = 0.631	5.9	0.617
Glow-worm		-1.7 *p* = 0.003	-1.0 *p* = 0.155	-2.1 *p* = 0.001	48.2	< 0.001

The first appearance dates of some of the five species were significantly correlated with one another, but the magnitude of these correlations suggests low levels of temporal synchrony (Table 3).

**Table 3:** Pearson correlations and p-values between the first appearance dates of the five invertebrates taken from the Houghton Fishing Club records in Hampshire, UK

	Grannom		Mayfly		Welshman’s Button		Mole Cricket	
	*r*	*p*	*r*	*p*	*r*	*p*	*r*	*p*
Mayfly	0.210	0.061						
Welshman’s Button	0.197	0.141	0.416	0.001				
Mole Cricket	0.387	0.042	0.253	0.211	0.198	0.321		
Glow-worm	0.443	0.008	0.495	0.003	0.409	0.018	0.317	0.100

## Discussion

In popular literature, river fly phenology is described in broad terms with actual dates rarely presented. Short-term observational or experimental studies (e.g. McCauley et al. 2015) on emergence broadly detect earlier emergence under warmer conditions but with exceptions (Finn et al. 2022). However, long-term observational studies appear to be sparse, likely reflecting the absence of data (Woods et al. 2022). Elliot (1996) reported a 30-year study on emergence of Alder fly *Sialis lutaria* in which emergence was advanced under warmer conditions, and advances in some Odonata have also been detected (Hassall et al. 2007). In the current paper we utilise the unique historical records from the Houghton Fishing Club which cover a century of observations from the southern UK. In addition, we have examined the phenology of two terrestrial invertebrates, for which phenological data are rare or absent (but see Atkins et al. (2016) for Glow-worm).

The appearance dates of Grannom, Mayfly and Welshman’s Button reported here are broadly consistent with Reisinger et al. (2010) who indicated the first, second and second halves of April, respectively, for first appearance. According to Atkins et al. (2016), the mean start of the glowing season of *Lampyris noctiluca* at four sites in southern England in 1998–2015 was around DOY 150, which would also match our results. We know of no other phenological data for Mole Cricket.

All three river flies were significantly related to Oxford air temperatures. A more local source of temperature data or water surface temperatures would have been more ideal. However, for the study period, data on air temperature are extremely limited and sources of water surface temperature, to the best of our knowledge, non-existent. Consequently, our broad temperature relationships should be taken to strongly suggest that river fly emergence dates have a temperature component. Fine detail observations of, *inter alia*, rain intensity, cloud cover, and sunshine may be necessary to further refine explanations of river fly emergence dates. However, such data are rarely available, and not at all for the study period.

Responses to air temperature were estimated to be between 3 and 6 days/°C for our three river flies, and of a similar magnitude for Glow-worm; broadly similar to those of affected Odonata reported by Hassel et al. (2007). No significant temperature response was found for Mole Cricket, suggesting the need for more appropriate climatic variables, such as soil temperatures where the larvae live and feed, since the 41-day range in first appearance indicates that an environmental driver is responsible.

We acknowledge the shortcomings of our data, the gaps in the record, the lack of more suitable local temperatures and the lack of standardisation in recording of appearance dates. However, despite these, significant temperature relationships were detected. Furthermore, contemporary descriptions of the River Test suggest it was not greatly affected by pollution or water extraction. We hope that this brief investigation will encourage others to examine any more recent data that exist for temperature responsiveness and change over time in a wider range of river fly species and locations.

## Data Availability

All data used are published and freely available from the sources indicated in the paper.
